# Different Drugs, Same End: Ultrastructural Hallmarks of Autophagy in Pathogenic Protozoa

**DOI:** 10.3389/fmicb.2022.856686

**Published:** 2022-03-29

**Authors:** Yasmin Pedra-Rezende, Isabela S. Macedo, Victor Midlej, Rafael M. Mariante, Rubem F. S. Menna-Barreto

**Affiliations:** ^1^Laboratório de Biologia Celular, Instituto Oswaldo Cruz, Fiocruz, Rio de Janeiro, Brazil; ^2^Laboratório de Biologia Estrutural, Instituto Oswaldo Cruz, Fiocruz, Rio de Janeiro, Brazil; ^3^Laboratório de Ultraestrutura Celular, Instituto Oswaldo Cruz, Fiocruz, Rio de Janeiro, Brazil

**Keywords:** protozoa, drugs, chemotherapy, autophagy, electron microscopy, endoplasmic reticulum profile, autophagosome, myelin-like structure

## Abstract

Protozoan parasites interact with a wide variety of organisms ranging from bacteria to humans, representing one of the most common causes of parasitic diseases and an important public health problem affecting hundreds of millions of people worldwide. The current treatment for these parasitic diseases remains unsatisfactory and, in some cases, very limited. Treatment limitations together with the increased resistance of the pathogens represent a challenge for the improvement of the patient’s quality of life. The continuous search for alternative preclinical drugs is mandatory, but the mechanisms of action of several of these compounds have not been described. Electron microscopy is a powerful tool for the identification of drug targets in almost all cellular models. Interestingly, ultrastructural analysis showed that several classes of antiparasitic compounds induced similar autophagic phenotypes in trypanosomatids, trichomonadids, and apicomplexan parasites as well as in *Giardia intestinalis* and *Entamoeba* spp. with the presence of an increased number of autophagosomes as well as remarkable endoplasmic reticulum profiles surrounding different organelles. Autophagy is a physiological process of eukaryotes that maintains homeostasis by the self-digestion of nonfunctional organelles and/or macromolecules, limiting redundant and damaged cellular components. Here, we focus on protozoan autophagy to subvert drug effects, discussing its importance for successful chemotherapy.

## Autophagy and Its Molecular Machinery

Autophagy is a physiological process of self-digestion of nonfunctional organelles and/or macromolecules, limiting redundant and damaged cellular components. This biochemical pathway can be selective or nonselective and guarantees eukaryotic homeostasis through the turnover and recycling of target cellular structures, which are pivotal events during cell growth and differentiation (Menna-Barreto, 2019; Abdrakhmanov et al., 2020; Klionsky et al., 2021). Despite nonselective characteristics during different conditions, such as starvation, numerous selective autophagic examples, including mitophagy, reticulophagy, and xenophagy (degradation by autophagy of mitochondria, endoplasmic reticulum, and pathogens, respectively), have been reported (Abdrakhmanov et al., 2020).

In pathological situations, including in protozoan infections, autophagy is increased to recover the cellular balance (Kirkegaard et al., 2004; Swanson, 2006). However, continuous induction of this pathway can culminate in autophagic cell death (Levine and Yuan, 2005). In protozoa, the autophagic phenotype is often induced *in vitro* by a great variety of drugs with different well-known mechanisms of action (Menna-Barreto et al., 2009c; Menna-Barreto, 2019).

Molecularly, autophagy is a highly conserved process regulated by autophagy-related genes (ATGs) previously identified in *Saccharomyces cerevisiae* (Klionsky et al., 2003), and their orthologs were subsequently described in all eukaryotes (Klionsky et al., 2021). Currently, three autophagic types are presented in the literature: macroautophagy (also called autophagy), microautophagy, and chaperone-mediated autophagy (CMA; Menna-Barreto, 2019; Abdrakhmanov et al., 2020).

Macroautophagy is characterized by the presence of double membrane organelles named autophagosomes that will address cellular material to be degraded in lysosomes. In a well-controlled process dependent on Atg proteins, a membrane structure (phagophore) surrounds damaged organelles or macromolecules, giving rise to autophagosomes (Shintani and Klionsky, 2004; Alvarez et al., 2008). The triggering of the process depends on the serine/threonine protein kinase TOR (target of rapamycin), a nutritional availability sensor, and Atg6 (beclin 1 in mammals), which is a phosphatidylinositol 3-kinase (PI-3K; Duszenko et al., 2011).

Unlike macroautophagy, autophagosomes are absent in microautophagy. The cellular material that will be degraded is engulfed by invagination of the lysosomal membrane. As demonstrated by electron microscopy, lysosomes full of small vesicles in their lumen are called multivesicular bodies. Unfortunately, this autophagic type is poorly studied due to the absence of specific markers (Menna-Barreto, 2019; Klionsky et al., 2021).

The most selective autophagic type is CMA, where signal pentapeptides (KFERQ, VDKFQ or QREFK) present in target proteins bind to cytosolic chaperones. The chaperone substrate binds to the lysosomal receptor LAMP-2A, promoting channel formation derived from receptor dimerization. Target molecules are degraded in the lysosomal lumen after entering through this channel (Duszenko et al., 2011).

Since the first description more than half a century ago, ultrastructural characterization remains a valuable tool for autophagic phenotype detection, allowing autophagosome identification without the use of specific markers (Menna-Barreto et al., 2009c; Menna-Barreto, 2019). More recently, knockdown or knockout of autophagic components strategies have also been commonly used. The gold-standard method for monitoring autophagic flux is the detection of Atg8 (LC3 in mammals) by morphological investigation after immunostaining microscopy (presence of LC3 puncta) and/or by immunoblotting (detection of LC3-I and LC3-II; Klionsky et al., 2021). In the present work, we reviewed different aspects of the protozoan autophagy exacerbation, discussing the possible role for drug resistance/susceptibility of these pathogens.

## Protozoan Diseases and Chemotherapy

### Chagas Disease

Chagas disease, which is caused by the protozoan *Trypanosoma cruzi*, is a neglected illness that affects approximately 6–7 million people worldwide, mostly in Latin America, and causes approximately 10,000 deaths per year (World Health Organization, 2020a). The occurrence of Chagas disease has also been reported in nonendemic countries, such as Canada, the United States, Australia, and Japan, due to the constant migration of individuals from endemic areas (Rassi et al., 2010). The transmission is mainly vectorial, depending on the infected triatomine bug, but *T. cruzi* can also be transmitted through blood transfusion, organ transplantation, ingestion of contaminated food or *via* transplacentary (Delgado and Gascón, 2020).

Clinically, Chagas disease presents two phases: acute and chronic. In the acute stage, despite patent bloodstream parasitemia, no specific symptoms are detected. In the chronic phase, individuals are asymptomatic in the indeterminate stage; approximately 30%–40% of cases progress to the symptomatic stage, which is characterized by cardiac and/or digestive alterations (Rassi et al., 2010). The clinical treatment of this disease is still based on the nitrocompounds benznidazole, and nifurtimox, which were discovered half a century ago and are highly effective in acute cases but exhibit limited efficacy in chronic patients (Table 1; Dias et al., 2016). Given limitations in currently available treatments, there is an urgent need for alternative and specific treatments. Several efforts have been directed to the development of new drugs or combinations for Chagas disease chemotherapy (Vannier-Santos et al., 2019).

**Table 1:** The main protozoal infections and their current chemotherapies.

Disease	Treatment	Mechanism of action	References
Chagas disease	Benznidazole Nifurtimox	Nitroreductases activation	Wilkinson et al., 2008
Sleeping sickness	Suramin	Glycosomal enzymes inhibition	Babokhov et al., 2013
	Pentamidine	Mitochondrial dysfunction	Vercesi and Docampo, 1992
	Eflornithine	Ornithine decarboxylase inhibition	LoGiudice et al., 2018
	Melarsoprol	Trypanothione inhibition	Kennedy, 2013
Leishmaniasis	Pentavalent antimonials	Sb (V) to Sb (III) reduction and type I DNA topoisomerases inhibition	Frézard et al., 2009
	Amphotericin B	Plasma membrane permeabilization and mitochondrial dysfunction	Lee et al., 2002
	Paromomycin	Protein synthesis inhibition	Kip et al., 2018
	Pentamidine	Mitochondrial dysfunction	Vercesi and Docampo, 1992
	Miltefosine	Cytochrome c oxidase inhibition	Luque-Ortega and Rivas, 2007
	Azolic compounds	CYP51 inhibition	Emami et al., 2017
Toxoplasmosis	Pyrimethamine and sulfadiazine	Block the parasite DNA synthesis (by inhibition of the folate metabolic pathway)	Dunay et al., 2018
	Spiramycin	Inhibits translocation (by interference in bacterial 50S ribosomal subunits)	Brisson-Noël et al., 1988; Brook, 1998
Malaria	Chloroquine	Intravacuolar pH increasing (hemoglobin digestion interfered)	Gabay et al., 1994
	Hydroxychloroquine	Intravacuolar pH increasing (hemoglobin digestion interfered)	Fox, 1993
	Artemether-lumefantrine	Free radical damage to parasite organelles and proteins.	White et al., 1999
	Atovaquone-proguanil	Mitochondrial electron transport inhibition	Painter et al., 2010; Vaidya, 2011
	Doxycycline	Inhibits apicoplast protein translation (organelle dysfunction)	Briolant et al., 2010
	Tetracycline	Protein synthesis inhibition (results in nonfunctional apicoplasts)	Dahl et al., 2006
	Clindamycin	Protein synthesis inhibition (results in nonfunctional apicoplasts)	Dahl et al., 2006
	Mefloquine	Intravacuolar pH increasing (hemoglobin digestion blockage)	Mungthin et al., 1998
	Artesunate	Parasite DNA damage	Gopalakrishnan and Kumar, 2015
Giardiasis Trichomoniasis and Amebiasis	Nitroimidazoles	Damage DNA and proteins	Lindmark and Müller, 1976; Leitsch et al., 2012; Muller et al., 2015;
	Benzimidazoles	Blocking glucose uptake and inhibit microtubules polymerization	Sears and O’Hare, 1988
	Nitazoxanide	Inhibition of enzymes that participates in energy conversion and possibly production of nitro radicals	Hoffman et al., 2007
	Paromomycin	Inhibition of protein synthesis	Edlind, 1989

### Sleeping Sickness

Caused by *Trypanosoma brucei*, sleeping sickness is a neglected disease transmitted by tsetse flies (*Glossina* genus) that occurs exclusively in sub-Saharan Africa with rural populations being more exposed to the vector (Centers for Disease Control and Prevention, 2019). At present, approximately 70 million people are at risk of infection, and 30,000 new cases are emerging regardless of disease control initiatives (World Health Organization, 2022). There are two subspecies that are pathogenic to humans: *T. brucei gambiense* and *T. brucei rhodesiense*. The most prevalent is *T. brucei gambiense*, which is present in western Africa and causes approximately 98% of reported cases, whereas *T. brucei rhodesiense* is found in eastern Africa and is much less prevalent (Centers for Disease Control and Prevention, 2019). Alternative routes of transmission have also been reported, such as transplacentary or mechanical transmission through other blood-sucking insects, but both are less frequent than the classical tsetse route (Drugs for Neglected Diseases Initiative, 2020).

Sleeping sickness presents in two distinct clinical phases depending on the localization of the parasite. In the first stage, *T. brucei* is mainly localized in the host bloodstream. However, in the second phase, the parasite is concentrated in the central nervous system, causing progressive neurological injury (Kennedy, 2013). The current treatment of the disease varies depending on the infectious species (*T. brucei gambiense* or *T. brucei rhodesiense*) as well as the disease phase (early or late stage). Pentamidine is the first choice for early stage *T. brucei gambiense* infection, while suramin is recommended for the early stage of *T. brucei rhodesiense* infection (Table 1; Kennedy and Rodgers, 2019). For the late stage, melarsoprol and eflornithine represent the primary treatment options, and the latter is generally used in association with nifurtimox. Fexinidazole is an oral treatment indicated as the first line for the first stage and nonsevere second stage in *T. brucei gambiense* (World Health Organization, 2022). The high toxicity of clinical drugs, especially melarsoprol, encourages the search for alternatives for anti-*T. brucei* chemotherapy.

### Leishmaniasis

Leishmaniasis is another neglected disease caused by 20 different *Leishmania* species that are spread by phlebotomine sandflies (Centers for Disease Control and Prevention, 2021a). Globally, more than 12 million people worldwide are infected, and 350 million people are at risk of infection with approximately 1.6 million new cases and 20,000–30,000 deaths each year (Pan American Health Organization, 2019). Distinct species of *Leishmania* spp. cause different clinical manifestations, and there are three different forms of the disease: mucosal, cutaneous, and visceral (kala-azar; Centers for Disease Control and Prevention, 2021a).

Due to the complexity of clinical manifestations and the diversity of etiological agent species, there are still many difficulties in finding a unique and effective treatment (Kaye and Scott, 2011). Pentavalent antimonial compounds constitute the first-line treatment, and meglumine antimoniate and sodium stibogluconate are the two main formulations. Other drugs, such as amphotericin B, paromomycin, pentamidine, miltefosine, and azolic compounds, are also commonly used alone or in combination (Table 1; Aronson et al., 2016). Undesirable side effects together with reports of resistance to conventional drugs justify the continuous search for new leishmanicidal agents (De Menezes et al., 2015). Among the novel approaches for cutaneous leishmaniasis, CO_2_ laser administration and thermotherapy, cryotherapy, electrotherapy, intralesional administration, combination therapy, immunomodulation, nanotechnology, and drug repurposing have been employed (Roatt et al., 2020).

### Toxoplasmosis

Toxoplasmosis is a disease caused by the obligate intracellular protozoan *Toxoplasma gondii*. This is the most prevalent infectious disease in humans, chronically infecting approximately one-third of the world’s population (Dubey, 2010). The successful worldwide distribution of *T. gondii* is attributed to the high diversity of host species it can infect, including almost all warm-blooded animals, and its multiple mechanisms of transmission, which include ingestion by the host of undercooked meat containing parasite cysts or oocyst-contaminated food or water (Dunay et al., 2018; Centers for Disease Control and Prevention, 2021b). Due to the infrequent or mild clinical manifestations, toxoplasmosis is considered an opportunistic infection in immunosuppressed patients and pregnant women, leading to severe symptoms, such as retinochoroiditis and mental disability (Centers for Disease Control and Prevention, 2021b).

Treatment of toxoplasmosis typically involves a combination of antimicrobials, such as pyrimethamine and sulfadiazine, plus folinic acid depending on the disease presentation, and particularities are noted in pregnant women (Table 1; Dunay et al., 2018; Centers for Disease Control and Prevention, 2021b). Unfortunately, the drugs used in clinical practice are only active against tachyzoites, the replicative form of the parasite, and do not demonstrate activity against tissue cysts containing bradyzoites, a latent stage of *T. gondii* that is present in the chronic phase of the disease (Dunay et al., 2018).

### Malaria

*Plasmodium* is the causative agent of malaria, a disease transmitted by the *Anopheles* mosquito, affecting tropical and subtropical countries, especially in Africa (Cowman et al., 2016; Centers for Disease Control and Prevention, 2021c). In 2019, malaria led to more than 400,000 deaths worldwide, mainly affecting children due to underdeveloped immunity. Thus, the disease is noted as one of the most serious and deadly illnesses in the world (Mbacham et al., 2019; World Health Organization, 2020b). Due to efficient public health strategies, an important reduction in cases can be observed in developed countries, and new cases are typically associated with immigrants and tourists from endemic areas (Cotter et al., 2013; Gachelin et al., 2018). Regarding clinical manifestations, malaria presents milder to more specific symptoms according to disease progression, including organ failure, blood abnormalities, cerebral malaria, and even death if not treated (Centers for Disease Control and Prevention, 2021c).

Treatment varies with the severity of the disease and the *Plasmodium* species, among other factors (White, 1996). Antimalarial drugs, such as chloroquine and hydroxychloroquine, have been the most widely administered to patients with uncomplicated malaria since their development. However, due to drug resistance over time, the disease can now be effectively treated with other drugs, such as artemether-lumefantrine (Table 1). For severe malaria, the patient should be treated with intravenous artesunate (Van Vugt et al., 2011; Centers for Disease Control and Prevention, 2021c).

### Giardiasis

*Giardia intestinalis* (syn. *Giardia lamblia*, *Giardia duodenalis*) is the most common parasite related to gastrointestinal infections in the world, affecting approximately 200 million people annually (Certad et al., 2017). Most cases are characterized by asymptomatic infections. However, these infections will release infectious cysts, perpetuating parasite dissemination (Capewell et al., 2021). The main symptoms of giardiasis are diarrhea and weight loss, which are linked to trophozoite adhesion to host intestinal epithelia, inefficient nutrient and water uptake, and triggering of an immune response (Leung et al., 2019). In some cases, giardiasis leads to a loss of barrier function and dysbiosis of the gut flora, presenting features similar to irritable bowel syndrome (Allain and Buret, 2020). The clinical effects of giardiasis are more significant in children, in which psychomotor and cognitive development can also be adversely affected during the infection, beyond the classical symptoms (Rogawski et al., 2017).

The current treatment of giardiasis is based on several drug classes. 5-Nitroimidazole derivatives are the most prescribed compounds, and metronidazole (MTZ) is often the drug of choice (Table 1). Among the other alternatives used, albendazole and mebendazole also stand out (Escobedo et al., 2016). MTZ presents undesirable side effects, usually resulting in treatment interruption. Drug resistance has been reported both *in vitro* and *in vivo*, and a complete parasitological cure has not been achieved (Argüello-García et al., 2020).

### Trichomoniasis

Despite the advances achieved from public campaigns carried out in recent decades, sexually transmitted infections (STIs) and their consequences are among the top five reasons that cause people in developing countries to seek medical treatment (Cudmore and Garber, 2010). Human trichomoniasis caused by the protozoan *Trichomonas vaginalis* is an STI with a wide geographic distribution that affects approximately 156 million people worldwide (Rowley et al., 2019). This disease is characterized by an infection of the urogenital tract and is more frequently noted in females. Severe and irritating inflammation resulting from exacerbated vaginal leukorrhea is the main pathological consequence (Cudmore and Garber, 2010; Secor, 2012). In men, trichomoniasis is typically asymptomatic, and the host acts only as a carrier. However, trichomoniasis can sporadically cause urethritis, prostatitis, and infertility (Secor, 2012). The greater predisposition of infected individuals to viral, bacterial, and fungal infections as well as the association between the presence of the parasite and a higher incidence of cervical cancer and an aggressive type of prostate cancer underscore the importance of trichomoniasis in human medicine (Hirt, 2013).

Trichomoniasis is only treated in women, and treatment is mainly based on MTZ administration (Table 1; Van Gerwen and Muzny, 2019). In addition to MTZ, tinidazole (TIN) is also prescribed due to better absorption and fewer gastrointestinal side effects than MTZ (Viitanen et al., 1985). Other drugs, such as disulfiram and nithiamide, can be used when patients have hypersensitivity to 5-nitroimidazoles (Sears and O’Hare, 1988).

### Amebiasis

Amebiasis is an enteric infection quite similar to giardiasis with one important difference: its disease state can range from intestinal inflammation to a severe liver abscess (Shirley et al., 2018). In humans, the disease is mainly caused by the nonflagellated protozoa *Entamoeba histolytica* (Shimokawa et al., 2012). Amebic infection is one of the main causes of diarrhea worldwide, mainly in young children. In developing countries, childhood diarrhea is a very common cause of death, accounting for approximately 9% of deaths in children under 5 years old (United Nations International Children’s Emergency Fund, 2018).

The most effective treatment for amebiasis is based on the administration of MTZ, mainly for the invasive disease form (Farthing, 2006). Paramomycin and diloxanide furoate are luminal agents commonly used to eliminate cysts from the colon (Zulfiqar et al., 2021). Other nitroimidazole derivatives, including tinidazole and ornidazole, are also used (Table 1; Chacín-Bonilla, 2013). The severe complication of amebiasis, namely, liver abscess, can be managed through aspiration using computed tomography as a guide combined with MTZ. In some cases, surgery is also required to treat gastrointestinal bleeding, megacolon, liver abscesses, and other severe damages when drainage is not possible (González-Alcaide et al., 2017).

## Autophagy in Protozoa

**Figure 1:**
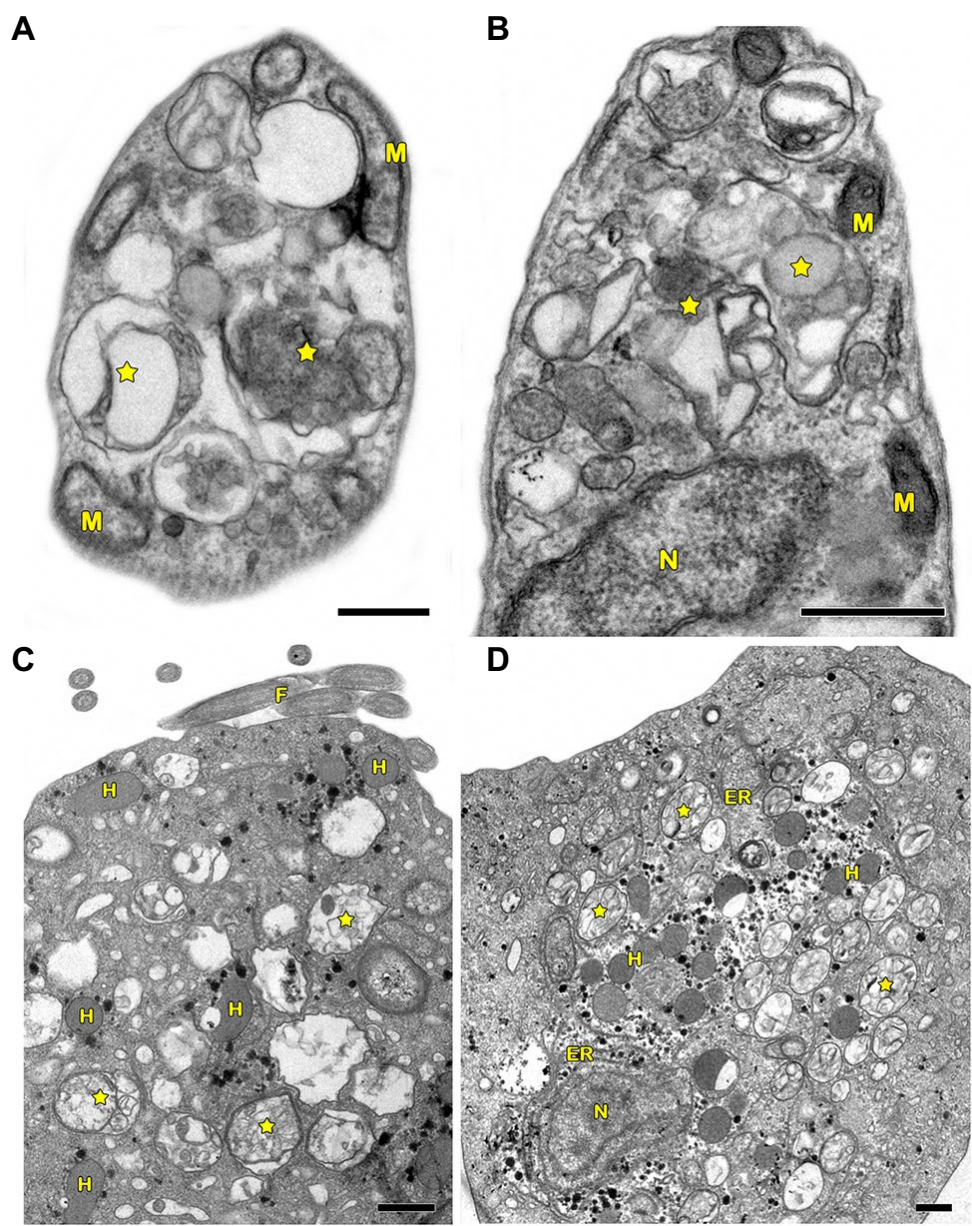
Transmission electron microscopy analysis of autophagy in pathogenic protozoa. **(A,B)** *Trypanosoma cruzi*. **(C,D)** *Trichomonas vaginalis*. **(A–D)** Under autophagic stimuli (drugs, starvation among others), parasites present a high number of autophagosomes (stars) distributed all over the cell. N, nucleus; M, mitochondrion; F, flagella; H, hydrogenosome; and ER, endoplasmic reticulum. Bars = 0.5 μm.

**Figure 2:**
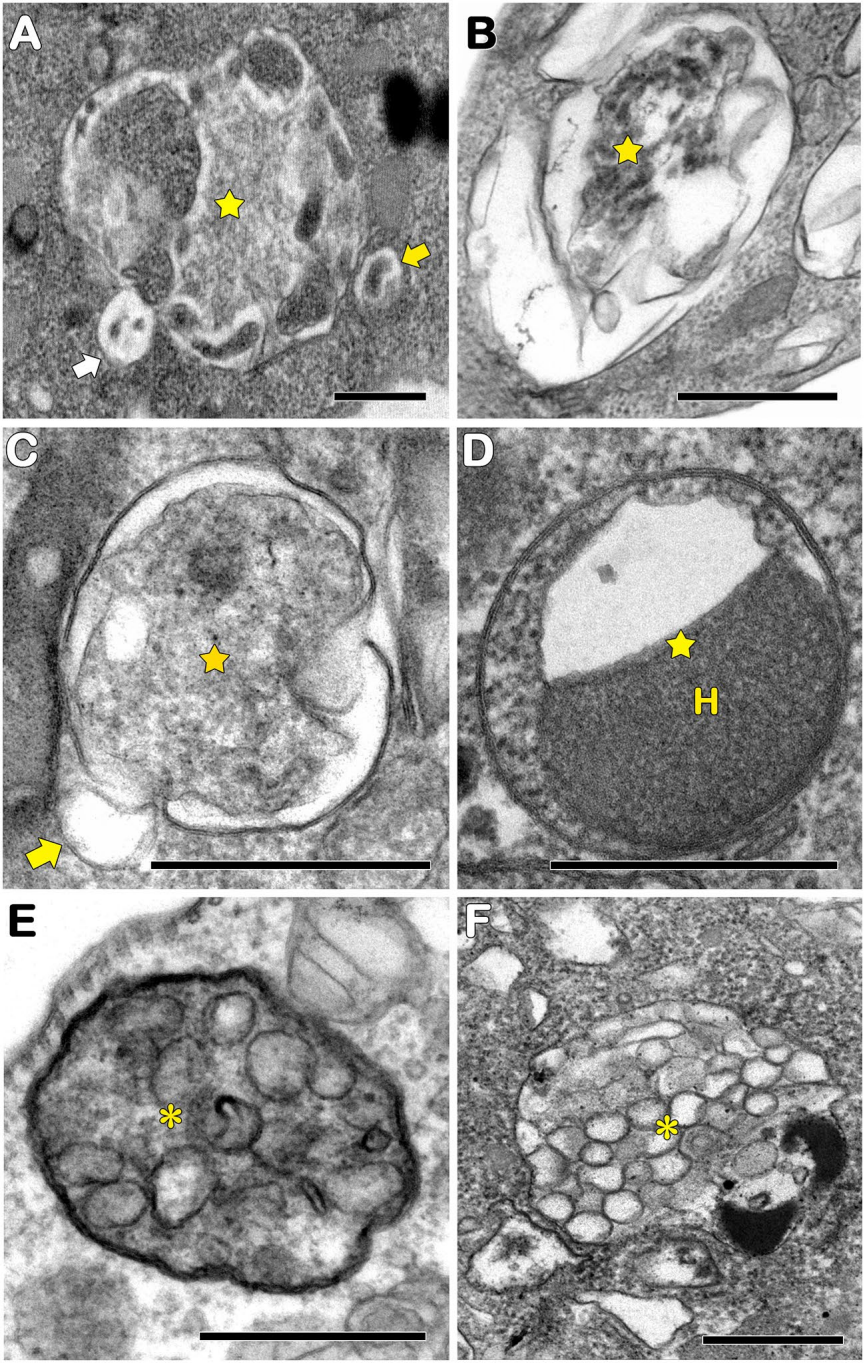
Transmission electron microscopy analysis of autophagosomes in pathogenic protozoa. **(A–C,E)** *Trypanosoma cruzi*. **(D,F)** *Trichomonas vaginalis*. **(A–D)** Autophagosomes with cargo in different levels of degradation (stars). Small vesicles in close contact with autophagosomal membrane were also observed (arrows). **(E,F)** Multivesicular bodies (asterisks). H, hydrogenosome. Bars = 0.5 μm.

**Figure 3:**
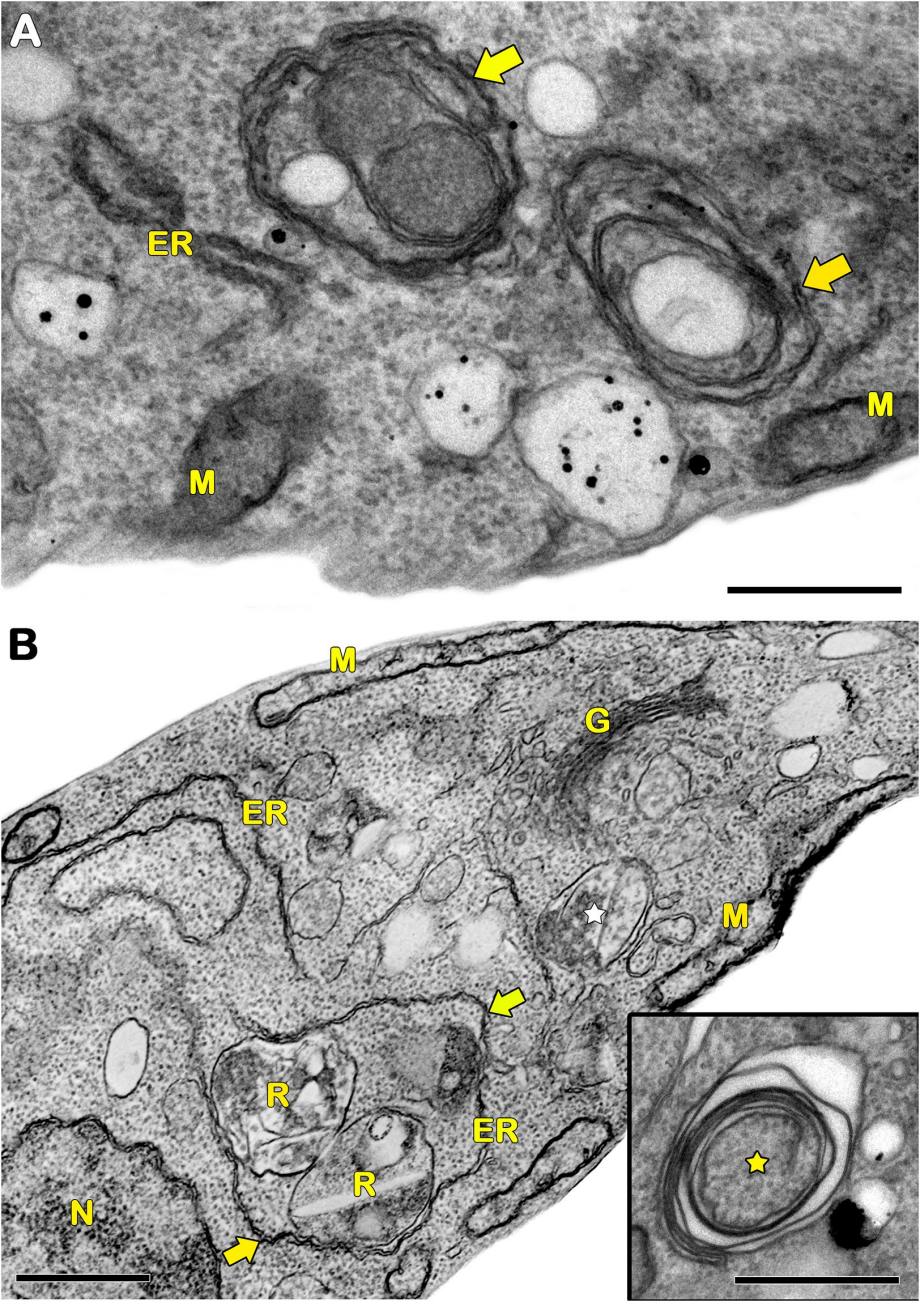
Transmission electron microscopy analysis of ER surrounding organelles in *Trypanosoma cruzi*. **(A,B)** Endoplasmic reticulum (ER) profiles is recurrently observed in close contact with a great variety of organelles (arrows) in treated parasites. The treatment with drugs also induces the appearance of concentric membrane structures (i.e., myelin-like structures) and the formation of autophagosomes (star). M, mitochondrion; N, nucleus; G, Golgi; and R, reservosome. Bars = 0.5 μm.

In protozoa, autophagy was first reported in *T. brucei* in 1977 by Vickerman and Tetley (1977) based on ultrastructural evidence. To date, parasites under starvation and/or subjected to other stress conditions commonly present autophagic features, including an increase in autophagosome number, multivesicular bodies, and myelin-like structures (Figures 1A–D, 2A–F, 3A,B; Benchimol, 1999; Maia et al., 2007; Corrêa et al., 2009; Menna-Barreto et al., 2009c; Besteiro et al., 2011; Ghosh et al., 2012; Koh et al., 2015; Picazarri et al., 2015; Souto et al., 2016; Nguyen et al., 2017b; Hernández-García et al., 2019; Araujo-Silva et al., 2021; Wu et al., 2021; Zhang et al., 2021). Interestingly, concentric membrane and myelin-like structures share morphological similarities to the phagophore described in yeast and mammals. The endoplasmic reticulum (ER), the main source of the phagophoric membrane, is frequently found surrounding degraded subcellular structures, especially in stressed parasites (Figures 3A,B; Maia et al., 2007; Corrêa et al., 2009; Martins-Duarte et al., 2009; Menna-Barreto et al., 2009a; Duszenko et al., 2011; Busatti et al., 2013; Hernández-García et al., 2019).

**Figure 4:**
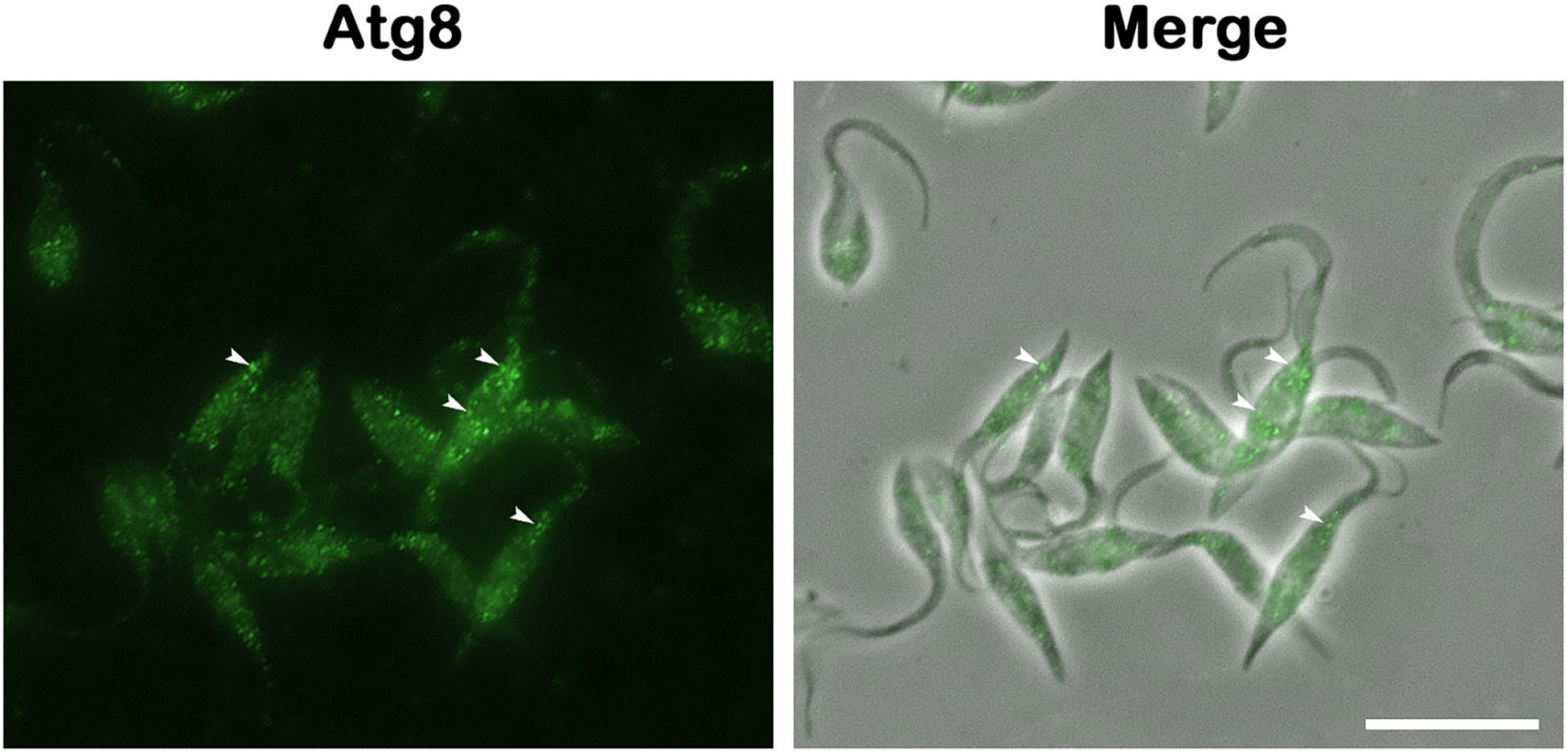
Atg8 detection is the gold standard method for monitoring autophagy. Rabbit anti-TcAtg8 antibody was employed to reveal Atg8 puncta (arrowheads) in *Trypanosoma cruzi* epimastigotes. Secondary antibody: anti-rabbit Alexa 488. Bar = 10 μm.

Regarding the molecular machinery, the autophagic pathway is well conserved among eukaryotes. Many ATG homologs have been identified in pathogenic protozoa, but some components are lacking or differ from those found in yeast. In trypanosomatids, genes involved in phagophore elongation and degradation of autophagosome cargo were detected by *in silico* approaches, including the complete Atg8 conjugation system (Atg3, Atg4, Atg7, and Atg8; Figure 4; Herman et al., 2006). Similar data were obtained in *Entamoeba* and *T. vaginalis*, where the Atg8 conjugation system was described, and the Atg12 complex is lacking (Picazarri et al., 2015; Hernandéz-García et al., 2019; Huang et al., 2019). In *Giardia*, bioinformatic analysis revealed the TOR, S6K1, PI3K, Atg1, Atg16, Atg7, Atg8, and Atg18 genes (Cernikova et al., 2020; Wu et al., 2021). *Toxoplasma gondii* seems to have well-conserved autophagic machinery, presenting several putative orthologs of yeast Atgs, including proteins of the Atg1, TOR, and PI3K complexes and Atg9 and Atg8/Atg12 systems (Lévêque and Besteiro, 2016; Besteiro, 2017). On the other hand, the Atg repertoire in *Plasmodium* did not reveal the presence of Atg24, TOR kinase, Atg9, Atg6, and Atg16, but the other Atgs found in *T. gondii* are also present (Lévêque and Besteiro, 2016; Besteiro, 2017).

The first functional analysis of Atgs in protozoa was performed in *L. major* in 2006 (Besteiro et al., 2006). The role of Atg8 and Atg4 was assessed in *T. cruzi* in 2008, and its activity was found to be directly related to the differentiation process of the parasite. The isoforms of Atg8 (TcAtg8.1 and TcAtg8.2) and Atg4 (Atg4.1 and Atg4.2) were investigated, showing the localization of Atg8.1 in autophagosomes of parasites under nutritional deprivation (Alvarez et al., 2008). Other Atg8 isoforms were identified in *Leishmania* spp. and *T. brucei* (Atg8, Atg8A, Atg8B, and Atg8C) and are regulated by the same two Atg4 isoforms (Rigden et al., 2005; Koopmann et al., 2009; Williams et al., 2009). On the other hand, in trypanosomatids, the Atg12 conjugation system is incomplete, and ATG5, ATG10, and ATG12 are lacking (Herman et al., 2006; Kiel, 2010). In *E. histolytica*, no Atg regulation was described during starvation, but EhAtg8 has a function in driving phagosome acidification (Picazarri et al., 2015). Atg8 is upregulated during the *G. intestinalis* differentiation process, and its expression induces parasite encystation (Wu et al., 2021). Moreover, *T. vaginalis* expresses two Atg8 genes, TvAtg8a and TvAtg8b, both of which retain a functional domain of Atg8. Moreover, TvAtg8a is more highly expressed (Huang et al., 2019).

The presence of genes encoding TOR kinases (TOR1 and TOR2) and their respective complexes (TORC1 and TORC2) was also detected in pathogenic trypanosomatids; however, their functions were not fully investigated. Following treatment with rapamycin, a classical autophagic inducer, an increased number of autophagosomes derived from TORC2 inhibition in *T. brucei* were observed, blocking parasite replication (Barquilla et al., 2008; Denninger et al., 2008). In *Giardia* and *Trichomonas*, rapamycin increased the number of Atg8 puncta, which may regulate parasite growth and differentiation into cysts (Hernandéz-García et al., 2019; Wu et al., 2021). A putative ortholog of mammalian TOR kinase is present in the *T. gondii* genome (TgTOR, TGME49_116440). To assess whether TgTOR is a component of the amino acid sensing mechanism, the parasites were treated with rapamycin, mimicking amino acid deprivation. Increasing drug concentrations induced a dose-dependent accumulation of Atg8 puncta, suggesting an increase in autophagic activity in the parasites (Besteiro et al., 2011) and a dose-dependent fragmentation of the parasite’s mitochondria (Ghosh et al., 2012).

As previously mentioned, autophagy seems to be particularly important for the success of the protozoan life cycle, especially during the differentiation steps (Alvarez et al., 2008; Besteiro et al., 2011; Lévêque et al., 2015; Picazarri et al., 2015; Smith et al., 2021; Wu et al., 2021). *Trypanosoma cruzi* epimastigotes are submitted to limited nutrients after their migration to the triatominae rectum, a crucial step for the occurrence of metacyclogenesis (Alvarez et al., 2008; Duszenko et al., 2011). Recently, Losinno et al. (2021) described the involvement of acidocalcisomes during this differentiation process. Autophagic vesicles released from this organelle fuse to reservosomes, contributing to reservosomal acidification and consequently increasing the hydrolytic activity of the cysteine protease cruzipain, resulting in parasite self-proteolysis (Losinno et al., 2021). Further experiments on the role of this self-processing and cruzipain activation in parasite differentiation and infection in both invertebrate and vertebrate hosts must be performed. In *Leishmania* spp., metacyclogenesis is also regulated by autophagy (Besteiro et al., 2006; Williams et al., 2006). Atg4.2 deletion blocked autophagic flux, leading to the accumulation of lipidated Atg8 and a decrease in the percentage of promastigotes under differentiation (Schoijet et al., 2017) as well as during *L. mexicana* amastigogenesis (Williams et al., 2006). In this parasite, megasomes are lysosome-like organelles that play a crucial role during the differentiation process. The deletion of two megasomal cysteine peptidases (CPA and CPB) also led to the impairment of amastigogenesis, and a high number of autophagosomes were found in mutant parasites (Williams et al., 2006; Kiel, 2010). At least for trypanosomatids, the involvement of autophagy in the regulation of virulence and infectivity in vertebrate hosts is clear (Besteiro et al., 2006), but the related molecular processes still need to be elucidated.

Due to the remarkable differences among vertebrate and invertebrate hosts, it is common sense that nutrient availability, temperature, and pH, among other environmental conditions influence protozoan metabolism, including ATP production (Kiel, 2010). Interestingly, the regulation of some of these metabolic adaptations occurs by autophagy. Li and He (2014, 2017) showed that autophagic flux participates in acidocalcisome acidification and that the blockage of organelle biogenesis also impairs the autophagic pathway in *T. brucei*. In *G. intestinalis*, Atg8 interacts with other proteins, such as myeloid leukemia factor (MLF) and FYVE domains, participating in the protein metabolism pathway and processing mitosomal and encystation proteins (Wu et al., 2021). In *Entamoeba*, *Eh*Atg8 is involved in the incorporation, scavenging and intracellular trafficking of nutrients (Picazarri et al., 2015). On the other hand, glucose restriction in *T. vaginalis* induces autophagy associated with TvAtg8 expression and autophagosome-like formation (Huang et al., 2019).

In apicomplexan parasites, Atg8 exerts unique functions. In addition to its cytosolic or, in the case of stressful conditions, vesicular location, it also localizes to the apicoplasts of *Toxoplasma* and *Plasmodium* (Kitamura et al., 2012; Kong-Hap et al., 2013; Tomlins et al., 2013; Lévêque et al., 2015). In *T. gondii*, Atg8 is responsible for the proper segregation of the organelle by tethering it to the centrosomes during the replication of the parasite (Lévêque et al., 2015). In *Plasmodium*, the protein contributes to apicoplast formation and maintenance (Tomlins et al., 2013). Given that the apicoplast is important for the synthesis of isoprenoid precursors and fatty acids, which are essential for parasite survival (Sheiner et al., 2013), Atg8 has an indirect but important role in apicomplexan metabolism.

Autophagy is also involved in the control of mitochondrial functionality and phospholipid homeostasis in protozoa (Besteiro et al., 2006; Williams et al., 2012; Vanrell et al., 2017). *Trypanosoma cruzi* epimastigotes submitted to an acidic environment or nutritional deprivation showed intense autophagic activity, ROS generation, and mitochondrial remodeling. These conditions reproduce the triatomine rectum environment, which is crucial for differentiation to the metacyclic form, suggesting a direct correlation between autophagy and mitochondrial remodeling during the process. On the other hand, insect blood digestion promotes transitory alkaline conditions. Our group also demonstrated that alkaline medium led to early exacerbation of autophagy and mitochondrial impairment. These features recovered over time, indicating a survival mechanism to increase the autophagic flux for the removal of damaged structures (Pedra-Rezende et al., 2021). *Leishmania major* promastigotes deficient in Atg5, which are not capable of forming autophagosomes, also presented a remarkable decrease in their virulence *in vitro* and *in vivo*. Strong mitochondrial dysfunction was also observed in these mutants together with the increased phosphatidylethanolamine (PE) content and ROS production, suggesting a conjugation of mitochondrial PE to Atg8 for autophagosome biogenesis (Williams et al., 2012). In *T. gondii*, starvation leads to mitochondrial dysfunction with consequent impairment of host cell invasion capacity and accumulation of Atg8 puncta (Besteiro et al., 2011; Ghosh et al., 2012). The use of mutated and conditional knockout parasites revealed that Atg8 and Atg3 are essential for Atg8 lipidation and autophagosome formation (Besteiro et al., 2011).

Regarding the selectivity of the autophagic pathway, selective degradation of *T. brucei* glycosomes (peroxisome-like) was proposed during the differentiation from bloodstream trypomastigotes into procyclic forms. This target organelle is crucial for parasite survival and is involved in bioenergetic metabolism and antioxidant defenses, corroborating the pivotal role of pexophagy in this trypanosomatid (Herman et al., 2008; Brennand et al., 2015).

**Figure 5:**
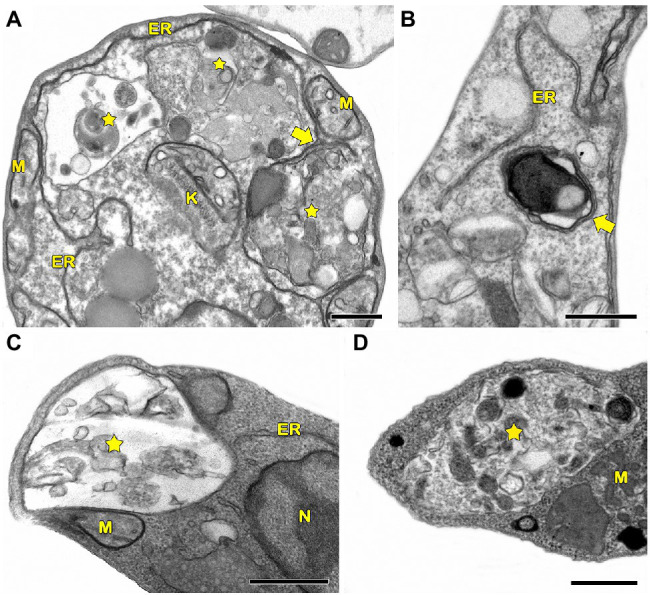
Transmission electron microscopy analysis of autophagic phenotypes conserved in pathogenic trypanosomatids. **(A,C)** *Leishmania braziliensis*. **(B,D)** *Trypanosoma cruzi*. **(A–D)** Both parasites treated with drugs showed similar autophagic features such as the presence of autophagosomes (stars) and the formation of endoplasmic reticulum (ER) surrounding organelles (arrows). M, mitochondrion; K, kinetoplast; and N, nucleus. Bars = 0.5 μm.

**Figure 6:**
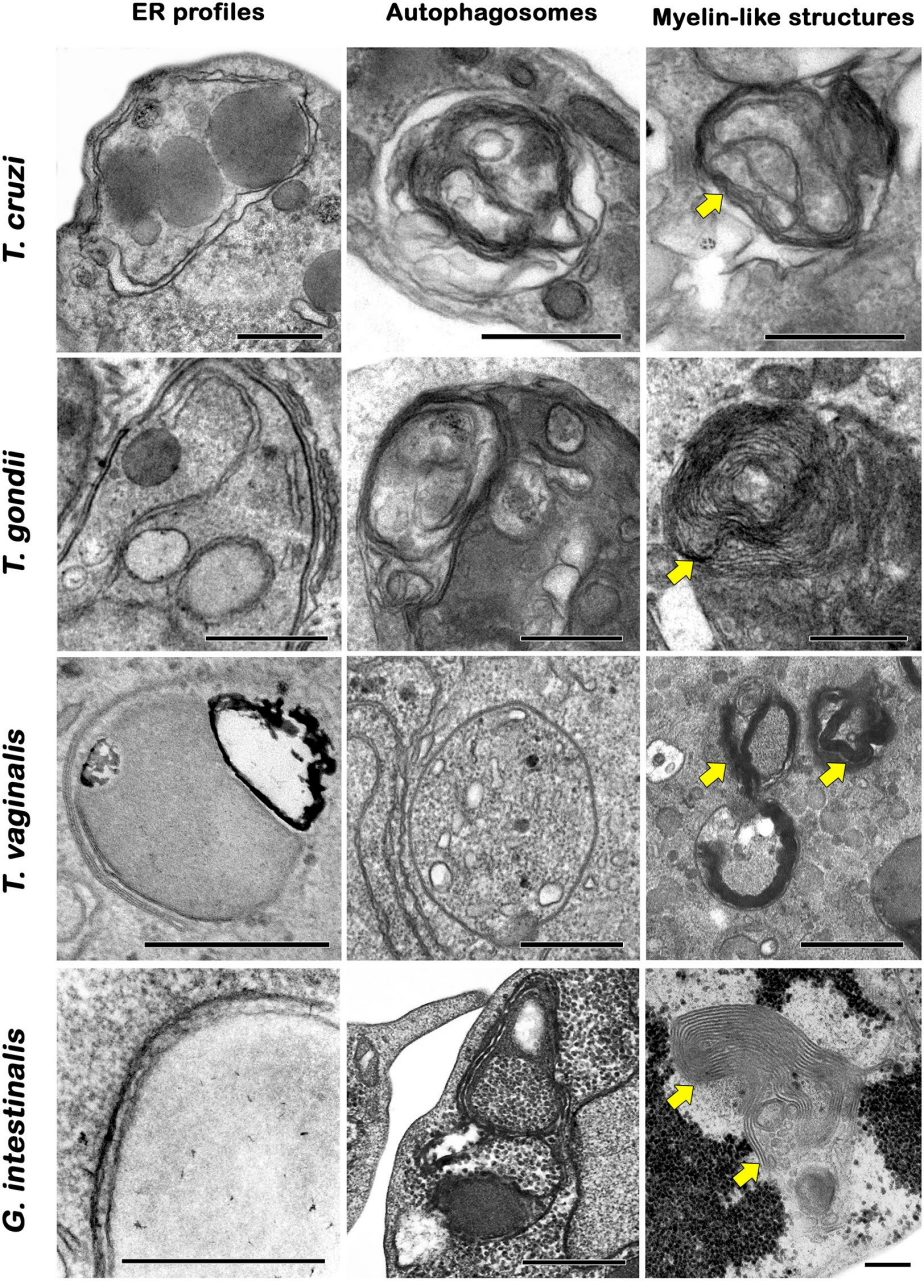
Transmission electron microscopy analysis of autophagic phenotypes conserved in protozoan parasites. Trypanosomatids, apicomplexans, trichomonadids, and diplomonadids share similar ultrastructural features of autophagy such as endoplasmic reticulum (ER) surrounding organelles, the formation of autophagosomes and myelin-like structures (arrows). Bars = 0.5 μm.

In general, autophagic phenotypes are very conserved, at least in pathogenic protozoans. Ultrastructural evidence (autophagosome formation, myelin-like structures and ER surrounding organelles) and Atg expression are frequently assessed in parasites under autophagic stimuli (Figures 5A–D, 6).

## Autophagic Phenotypes in Drug-Treated Parasites

Following the recent guidelines for monitoring autophagy, autophagic phenotypes in protozoa after treatment with drugs were assessed by different techniques, including Western blotting and electron and fluorescence microscopy (Klionsky et al., 2021). Innumerous compounds from distinct classes induced different autophagic phenotypes (to different degrees) in protozoan parasites.

One of the most frequent morphological features of autophagy identified in these parasites is the presence of concentric membrane structures or myelin-like structures. Ultrastructural studies noted this structure in protozoa treated with all classes of compounds (Vannier-Santos et al., 1995; Benchimol, 1999; Braga et al., 2005; Granthon et al., 2006; Uzcátegui et al., 2007; Menna-Barreto et al., 2009a,b; Carvalho et al., 2010; Bombaça et al., 2018, 2021; Martínez-García et al., 2018; Araujo-Silva et al., 2021).

The increased number of autophagosomes, a determinant of macroautophagy occurrence, is another very recurrent phenotype detected in treated protozoa (Braga et al., 2005; Granthon et al., 2006; Uzcátegui et al., 2007; Dos Santos et al., 2010; Schurigt et al., 2010; Besteiro et al., 2011; Sengupta et al., 2011; Fernandes et al., 2012; Ghosh et al., 2012; Veiga-Santos et al., 2013; Souto et al., 2016; Nguyen et al., 2017b; Scariot et al., 2017; Lim et al., 2018; Martínez-García et al., 2018; Hernandéz-García et al., 2019; Huang et al., 2019; de Paula et al., 2020; Silva et al., 2020; Nishi et al., 2021; Zhang et al., 2021).

As previously mentioned, Atg8 is considered the gold standard method for autophagic evaluation. Treatment of *L. donovani* with cryptolepine induced an increase in the number of Atg8 puncta (Sengupta et al., 2011). Similar findings were observed in *T. brucei* treated with L-leucine methyl ester and bacteriocin AS-48 (Koh et al., 2015; Martínez-García et al., 2018). In *T. gondii*, starvation or treatment with the drug monensin or some antimalarial compounds induces a time-dependent accumulation of Atg8 puncta (Besteiro et al., 2011; Lavine and Arrizabalaga, 2012; Varberg et al., 2018). Similar results were obtained when parasites were treated with the ER stress-inducing agents dithiothreitol, brefeldin A, or tunicamycin (Nguyen et al., 2017a). Alternatively, the autofluorescent compound monodansyl cadaverine (MDC) is typically employed to assess autophagy, despite the nonspecificity of this marker. Several groups observed an increase in MDC labeling after the treatment of parasites with different compounds (Sengupta et al., 2011; Fernandes et al., 2012; Lazarin-Bidóia et al., 2013; Veiga-Santos et al., 2013; Desoti et al., 2014; Scariot et al., 2017, 2019; de Paula et al., 2020; Bortoleti et al., 2021; Nishi et al., 2021; Zhang et al., 2021).

ER profiles surrounding cytoplasmic structures and organelles are also commonly detected in treated protozoa. Fernandes et al. (2012) demonstrated that treatment with triazolic naphthoquinone led to the appearance of ER in close contact with reservosomes of *T. cruzi* epimastigotes. Especially in this case, the authors described the Golgi as an alternative source of the phagophoric membrane (Fernandes et al., 2012). In *L. amazonensis*, elatol and amiodarona induced a similar phenotype and pronounced swelling of the mitochondrion and destabilization of the plasma membrane (Dos Santos et al., 2010; Macedo-Silva et al., 2011). In *T. gondii*, treatment with thiolactomycin analogs induced dramatic morphological changes in parasite shape and intracellular organization, including abnormal amounts of concentric membranes expanded throughout the parasite cytoplasm, possibly representing ER profiles (Martins-Duarte et al., 2009).

To evaluate the specific role of autophagy in a drug mechanism of action, one of the most common experimental protocols is the treatment of parasites with autophagic inhibitors, such as wortmannin and 3-MA (Pasquier, 2016; Klionsky et al., 2021). In *T. cruzi*, the use of these inhibitors completely abolished the trypanocidal effect of naphthoimidazoles, reinforcing autophagy as part of the mechanism of cell death induced by these compounds (Menna-Barreto et al., 2009a). Similar results were obtained with *T. gondii*, where preincubation with 3-MA efficiently abrogated the effects of monensin on mitochondrial fragmentation (Lavine and Arrizabalaga, 2012). Interestingly, 3-MA was not able to prevent antimalarial compounds from the medicines for malaria venture malaria box MMV2- or MMV3-induced mitochondrial disruption, indicating that autophagy may occur downstream of mitochondrial fragmentation *via* PI3K-independent mechanisms (Varberg et al., 2018).

## Concluding Remarks

Protozoan diseases still represent a significant challenge, demanding specific public health strategies, especially in low-income countries. Many of them are considered neglected diseases that impair physical and cognitive development, limiting individual productivity and resulting in economic issues (World Health Organization, 2015; Centers for Disease Control and Prevention, 2021d). Different parasite forms from a great variety of subpopulations also contribute to the increase in the drug resistance of pathogens, reinforcing the necessity of a continuous search for alternative compounds with anti-protozoan activity. During preclinical tests, cellular, molecular, and biochemical information about the targets of the novel compounds is critical for the characterization of drug safety and specificity.

Indeed, a detailed description of the mechanisms of action is still lacking, even for clinical drugs. As an example, the trypanocidal actions of benznidazole and nifurtimox, which are employed for Chagas disease treatment, are still not completely understood more than 50 years after their discovery (Menna-Barreto and De Castro, 2016). Furthermore, many new compounds with potent anti-protozoan activity do not exhibit predicted biological effects (Chatelain and Ioset, 2011; Gaspar et al., 2015), emphasizing the importance of the identification of effective molecular targets. Despite multidisciplinary efforts involving high-throughput screenings to discover novel candidates for the treatment of protozoan diseases (Annang et al., 2015; Peña et al., 2015), few studies have elucidated their mechanisms of action. One of the main reasons is the lack of efficient and practical tools to assess molecular drug targets *in vitro* and *in vivo*.

Electron microscopy was extensively employed in the first identification of primary drug targets in treated parasites, such as organelles and cellular structures (Corrêa et al., 2009; Menna-Barreto and De Castro, 2016). Ultrastructural analysis may allow inferences about the action of the compounds. Many studies also suggest the triggering of cell death processes as part of the drug mechanism (Maia et al., 2007; Corrêa et al., 2009; Menna-Barreto et al., 2009a,b; Menna-Barreto, 2019). Despite all ultrastructural evidence, the occurrence of programmed cell death in protozoan parasites is very controversial due to the absence of precise information about biochemical and molecular events, especially those involved in regulatory processes (Menna-Barreto, 2019). Until the convincing identification of the executioners, these phenotypes should be classified as unregulated processes or incidental necrosis (Proto et al., 2013; Menna-Barreto, 2019).

**Figure 7:**
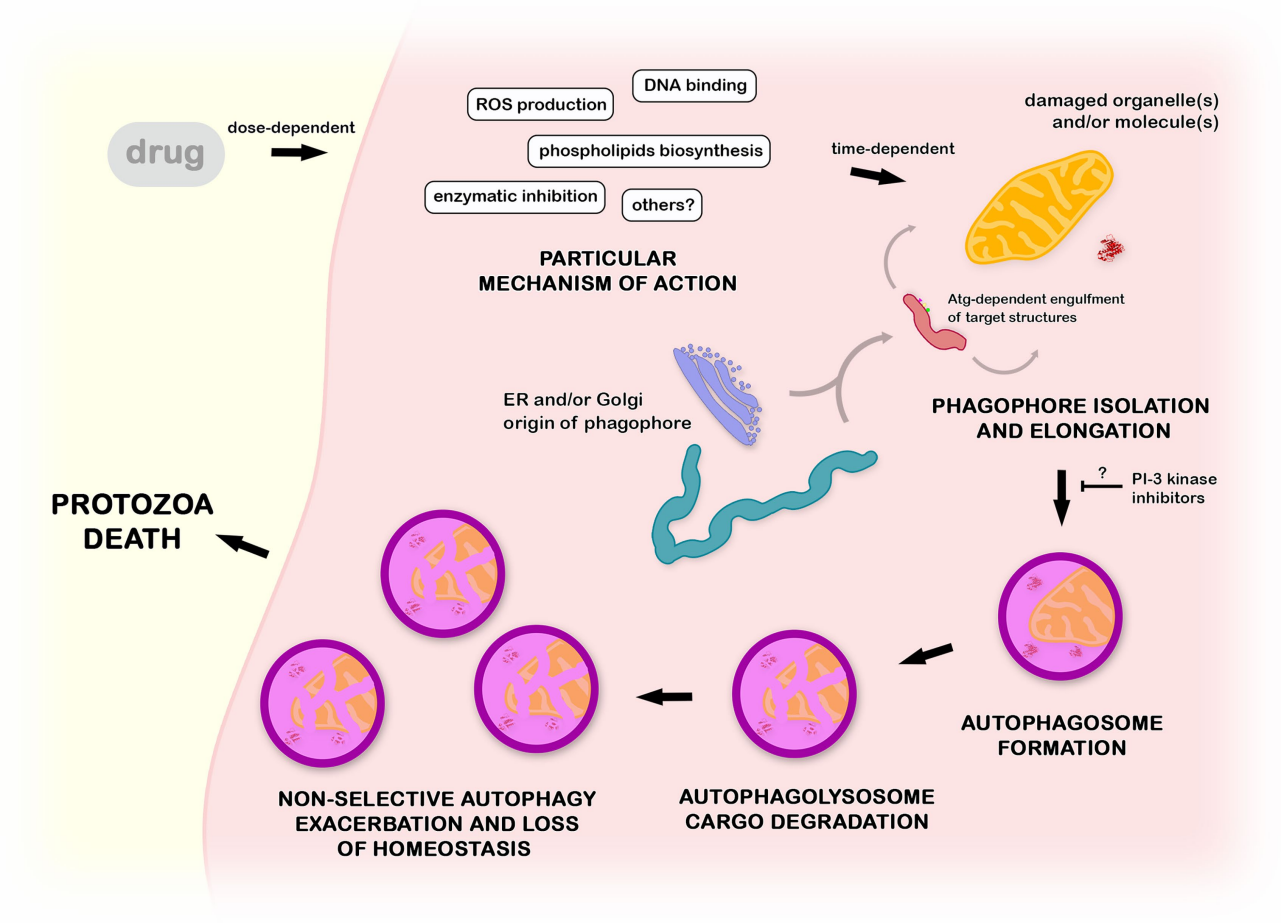
Mechanistic proposal of autophagy in the mode of action of anti-protozoa drugs. Compounds with distinct mechanisms of action lead to the impairment of cellular structures such as organelles and macromolecules. Such damaged structures are randomly engulfed by a phagophore in a Atg-dependent process, forming an autophagosome. After the fusion with lysosome, the cargo is degraded inside the autophagolysosome. The non-selective continuous autophagic exacerbation promotes the breakage of the protozoa homeostasis, culminating in an accumulation of a high number of autophagic vacuoles (autophagosomes and/or autophagolysosomes) and consequent autophagic cell death. This process could be at least partially inhibited by the pre-incubation with classical PI-3 kinase inhibitors as wortmannin or 3-methyladenine.

The treatment of different protozoa with distinct classes of drugs led to a convergent autophagic phenotype. Ultrastructural lesions in organelles, such as mitochondria, reservosomes, or hydrogenosomes, are usually closely associated with the appearance of endoplasmic reticulum profiles and an increase in the number of autophagosomes (Menna-Barreto et al., 2009a,b; Hernandéz-García et al., 2019). Together with the formation of myelin-like structures, these are the most recurrent autophagy-related phenotypes described (Figure 7). Based on morphological findings, the autophagic role in cell death has been postulated, but a description of the regulatory events involved is lacking (Bera et al., 2003; Baehrecke, 2005; Menna-Barreto et al., 2009a). It is well known that autophagy plays a pivotal role in homeostasis maintenance in eukaryotes, including protozoan parasites. Drugs impair different molecular pathways, causing the loss of the balance in the turnover of crucial cellular structures. Such an imbalance promotes an exacerbation of nonselective autophagy, culminating in nonspecific damage (Baehrecke, 2005). This is a reasonable explanation for the appearance of the autophagic phenotype in treated parasites regardless of the drug and/or mechanism of action involved (Menna-Barreto et al., 2009b; Menna-Barreto, 2019). In summary, autophagy represents survival machinery responsible for the removal of cellular structures damaged by compounds.

## Author Contributions

RM-B conceived the work and drafted the manuscript. YP-R and RM-B wrote trypanosomatids subjects. RM and IM wrote the apicomplexan part. VM wrote about anaerobic parasites. All authors contributed to the article and approved the submitted version.

## Funding

This work was supported by CNPq, CAPES, FAPERJ, and FIOCRUZ.

## Conflict of Interest

The authors declare that the research was conducted in the absence of any commercial or financial relationships that could be construed as a potential conflict of interest.

## Publisher’s Note

All claims expressed in this article are solely those of the authors and do not necessarily represent those of their affiliated organizations, or those of the publisher, the editors and the reviewers. Any product that may be evaluated in this article, or claim that may be made by its manufacturer, is not guaranteed or endorsed by the publisher.
